# eHEALS: The eHealth Literacy Scale

**DOI:** 10.2196/jmir.8.4.e27

**Published:** 2006-11-14

**Authors:** Cameron D Norman, Harvey A Skinner

**Affiliations:** ^2^Faculty of HealthYork UniversityTorontoONCanada; ^1^Department of Public Health SciencesUniversity of TorontoTorontoONCanada and Peter A. Silverman Global eHealth Program

**Keywords:** Internet, literacy, public health, psychometrics, quantitative evaluation

## Abstract

**Background:**

Electronic health resources are helpful only when people are able to use them, yet there remain few tools available to assess consumers’ capacity for engaging in eHealth. Over 40% of US and Canadian adults have low basic literacy levels, suggesting that eHealth resources are likely to be inaccessible to large segments of the population. Using information technology for health requires eHealth literacy—the ability to read, use computers, search for information, understand health information, and put it into context. The eHealth Literacy Scale (eHEALS) was designed (1) to assess consumers’ perceived skills at using information technology for health and (2) to aid in determining the fit between eHealth programs and consumers.

**Objectives:**

The eHEALS is an 8-item measure of eHealth literacy developed to measure consumers’ combined knowledge, comfort, and perceived skills at finding, evaluating, and applying electronic health information to health problems. The objective of the study was to psychometrically evaluate the properties of the eHEALS within a population context. A youth population was chosen as the focus for the initial development primarily because they have high levels of eHealth use and familiarity with information technology tools.

**Methods:**

Data were collected at baseline, post-intervention, and 3- and 6-month follow-up using control group data as part of a single session, randomized intervention trial evaluating Web-based eHealth programs. Scale reliability was tested using item analysis for internal consistency (coefficient alpha) and test-retest reliability estimates. Principal components factor analysis was used to determine the theoretical fit of the measures with the data.

**Results:**

A total of 664 participants (370 boys; 294 girls) aged 13 to 21 (mean = 14.95; SD = 1.24) completed the eHEALS at four time points over 6 months. Item analysis was performed on the 8-item scale at baseline, producing a tight fitting scale with α = .88. Item-scale correlations ranged from *r* = .51 to .76. Test-retest reliability showed modest stability over time from baseline to 6-month follow-up (*r* = .68 to .40). Principal components analysis produced a single factor solution (56% of variance). Factor loadings ranged from .60 to .84 among the 8 items.

**Conclusions:**

The eHEALS reliably and consistently captures the eHealth literacy concept in repeated administrations, showing promise as tool for assessing consumer comfort and skill in using information technology for health. Within a clinical environment, the eHEALS has the potential to serve as a means of identifying those who may or may not benefit from referrals to an eHealth intervention or resource. Further research needs to examine the applicability of the eHEALS to other populations and settings while exploring the relationship between eHealth literacy and health care outcomes.

## Introduction

How do we determine whether individuals have the capacity to engage with eHealth programs and interventions? Health practitioners, eHealth developers, and researchers alike need to know if electronic health tools are suitable methods for effectively promoting population health and aiding health care. An often unmentioned assumption underlying the deployment of eHealth resources intended for public consumption is that consumers have the skills to use such resources to their optimal level. Yet, with over 40% of US and Canadian adults having basic (or prose) literacy levels below what is considered necessary to optimally participate in civil society [1,2], it is unlikely that eHealth will provide population-level benefits as it requires much more than just prose literacy. Consumer-directed eHealth requires the ability to seek out, find, evaluate and appraise, integrate, and apply what is gained in electronic environments toward solving a health problem, or *eHealth literacy* [3]. This composite skill requires that people are able to work with technology, critically think about issues of media and science, and navigate through a vast array of information tools and sources to acquire the information necessary to make decisions.

Informed decision making requires that people can adequately access, understand, and process health information to meet their needs. Access refers both to the literal ability to access information resources like health websites, but also the quality of this access. This includes the quality of the technology (eg, Internet connection speed, hardware, software) and the conditions of use, such as whether people have the privacy or time to properly engage eHealth resources. Access in the Internet age also requires an ability to derive meaning from text. As basic literacy skills rise, so does the ability to use computers effectively to solve problems, regardless of age, income, or education [4].

Given issues of access and literacy, health practitioners in clinical and public health settings require an understanding of what abilities their patients/clients have before recommending eHealth resources. This article describes the development and psychometric evaluation of a measure of eHealth literacy designed for broad use in supporting consumer eHealth in public health and clinical care.

### Health and Literacy in an Electronic Context

Health literacy has been identified as a public health goal for the 21st century and a significant challenge facing health care globally [5-7]. The recent Institute of Medicine report [8] on health literacy highlights the need to look at the different contexts where health information is obtained and used as part of a strategy of addressing health literacy. More than ever, this health information context includes electronic resources such as the World Wide Web and other technologies that now play an increasing role in consumer health [9,10]. Electronic health information introduces challenges pertaining to both the medium and the message that differ substantially from other media forms. Issues of access to information, retrieval, evaluation and appraisal, and other quality markers fundamentally differ in unregulated environments such as the Web, where new information is added every minute of every day. Being health literate in an electronic world requires a different or at least expanded set of skills to engage in health care and promotion, or eHealth literacy.

eHealth literacy is comprised of six core skills, or literacies: (1) traditional literacy, (2) health literacy, (3) information literacy, (4) scientific literacy, (5) media literacy, and (6) computer literacy [3]. The foundations of the eHealth literacy concept are based in part on social cognitive theory and self-efficacy theory [11], which promote competencies and confidence as precursors to behavior change and skill development and are described in detail elsewhere [3]. The challenge is developing the means to assess this skill in order to provide strategies to assist consumers in using eHealth to its fullest potential.

The eHealth Literacy Scale (eHEALS) has been developed to address the need to assess eHealth literacy for a wide range of populations and contexts. The eHEALS is a self-report tool that can be administered by a health professional and is based on an individual’s perception of her or his own skills and knowledge within each measured domain. The instrument is designed to provide a general estimate of consumer eHealth-related skills that can be used to inform clinical decision making and health promotion planning with individuals or specific populations.

It is not unreasonable to assume a link between eHealth literacy and technology use in general. The more an individual uses technology, the more likely they are to develop skills in using that technology as a tool. For that reason, youth can serve as an ideal group to test a measure of eHealth literacy given this population’s high familiarity with technology. In Canada, 99% of adolescents have access to the Internet, and the majority of Canadian teens report using the Internet for health in some capacity [12]. Although questions remain about the quality of this Internet access [13], this group is most likely to be familiar with information technology tools and is more likely to use eHealth than most other populations[13,14]. Despite having relative familiarity with eHealth, many adolescents are unable to derive the full benefit from it. Gray and colleagues looked at the issue of health literacy and technology in adolescents and found many teens experienced difficulty engaging with eHealth and understanding or using health information online, despite frequently using information technologies [15].

Regardless of the population of interest, the need to navigate the Internet with confidence is particularly important for health issues in which the consequences for using low quality, misleading, or false information are great [16]. By providing tools and resources to evaluate health information online and critically appraise eHealth resources, we offer an opportunity to both protect consumers from harm and empower them at the same time [17,18]. In order to provide relevant tools to aid consumers in navigating through eHealth, an understanding of what skills consumers possess at the outset, or their eHealth literacy, is required. This study’s objective is to develop and test a functional method of assessing perceived eHealth literacy skills to aid consumers and health practitioners alike in assessing a fit with eHealth to support clinical care and promote population health.

### Scale Development

A review of the literature was undertaken on each of the six key literacies that comprise Norman and Skinner’s eHealth literacy model [3] in the Medline, PsycInfo, ERIC, Sociological Abstracts, and Web of Science databases to identify existing literacy measures. Although some measures were found, few had been rigorously psychometrically evaluated, and some were designed for specific projects that were not relevant to how the literacy concept was conceived of in relation to the eHealth literacy model. Given these constraints, it was decided that creating items from scratch was appropriate. Based on the theoretical model, an initial item pool was established and an iterative process of item reduction was used to create an instrument that could be easily deployed within a variety of settings and contexts as intended. The initial battery was circulated by the investigator to colleagues working in the area of eHealth for comment and review. After this initial review, the eHEALS was given to youth involved with TeenNet Research [19] to test general readability, item wording, and relevance. Youth are a consumer group with developing literacy skills and thus were expected to reflect the reading needs of a lower literacy population. These youth ranged in age from 12 to 19, came from many different social, ethnic, and educational backgrounds, and represented diverse interests among the adolescent population. Reviews were conducted in small groups over the course of 3 months. Further readability tests were conducted during the pilot phase of the project described below. Revisions were made as necessary before being pilot tested with a larger number of participants.

### Pilot Testing

A total of 89 youth (ages 14-24) completed the initial, larger version of the eHEALS as part of a pilot test and provided comments on the readability and item wording in focus groups immediately following completion of the instrument in paper form. The eHEALS was subsequently reviewed and modified to create the final battery of 8 items based on the qualitative and response feedback from participants, theoretical fit, and comprehensiveness. This study represents its first full psychometric assessment.

## Methods

### Participants

This study was conducted as part of another larger evaluation project looking at eHealth smoking prevention and behavior change using a randomized controlled trial. The study described here involved participants from one arm of this trial given that the other arm was intended to promote eHealth literacy, thus potentially confounding the results of the psychometric review. The study recruited 664 adolescents from 14 secondary schools in a large Canadian city. Students in grades 9, 10, and 11 were sampled from a variety of class types encompassing different subject areas (eg, physical education, computer science) and formats (eg, single sex and mixed sex classes). An attempt was made to involve a cross-section of schools in the study through active recruitment directly with school administrators and teachers. Schools were offered a modest stipend for their involvement, but no direct incentives were provided to individual students as the study was considered a part of classroom activities due to a fit with the curriculum. Ethical approval for the study was obtained from the ethical review boards or committees from the University of Toronto, Toronto Public Health (a project partner), and both of the participating school boards.

### Sample Characteristics

Age of the participants ranged from 13 to 21 (mean = 14.95; SD = 1.24), which included recent immigrants who may have been older than typical students in a particular grade. Sex was unevenly distributed within the sample due in part to the involvement of many single-sex classes involved in the study (boys N = 370; girls N = 294). The sample reflected the ethno-cultural diversity of the community, with the most commonly identified ethno-cultural groups being of Eastern European (N = 107, 16% of sample), East Asian (N = 103, 16% of sample), and Central Asian origin (N = 78), while 16% (N = 106) of participants did not identify with a particular cultural group. Ethno-cultural identity was determined using categories modified from Statistics Canada [2], which categorize individuals based on sociocultural and geopolitical differences in addition to racial ones. Most participants, 39%, were in grade 9 (N = 260), 29% in grade 10 (N = 193), and 32% in grade 11 (N = 211).

### Technology Use

Participants reported being regular users of various forms of information technologies: 71% of participants (N = 468) reported using email at least once a week, with 37% (N = 544) using it daily; 79% (N = 522) reported using the Web each week, with 35% (N = 232) using it daily; and 71% (N = 473) of participants were regular (weekly) users of text messaging, with 42% (N = 280) reporting using it daily. Most participants reported that their primary access point for the Internet was at home (81%, N = 537); school (42%, N = 276) and friends’ homes (34%, N = 227) were identified as the most common secondary access points for the Internet.

### Procedure

The eHEALS was administered within a larger battery of measures as part of a combined randomized trial evaluation of an eHealth literacy promotion intervention and a Web-based smoking cessation program [20]. For the purposes of evaluating the eHEALS, data from the smoking prevention and cessation arm of the study (ie, the control condition) were used in the reliability testing of the eHEALS instrument. The eHEALS was administered using a pencil and paper survey delivered with other health measures used as part of a larger study. Participants completed the eHEALS prior to the intervention being delivered, immediately after the intervention, and at 3- and 6-month follow-up. Pre-test and immediate post-intervention data were collected during a single 75-minute class period.

### Data Analyses

Internal consistency reliability was assessed using SPSS version 11.5[21] using the SPSS RELIABILITY command. Reliability (item) analysis was used to examine differences between boys and girls. Factors were identified using the simple structure approach solution based on reported eigenvalues over 1.0 [22] using principal components analysis with SPSS FACTOR. This approach relies on a priori hypotheses to guide the selection of models, supported by scree tests and interpretability of the factor based on item/scale correlations. The results were considered using Comrey and Lee’s (1992) guidelines whereby factor loadings in excess of .71 (50% overlapping variance) were considered excellent, .63 (40% overlapping variance) very good, and .55 (30% overlapping variance) good [23]. Factor loadings lower than .55 were considered fitting if items or scales correlated on only a single factor.

## Results

### Internal Consistency and Factor Analysis

The internal consistency reliability and factor analysis results are presented in Table 1. Each item in the eHEALS uses a 5-point Likert scale to answer each question with response options ranging from “strongly agree” to “strongly disagree” (Multimedia Appendix 1). Item analysis was performed on the 8-items, producing a tight fitting scale with coefficient alpha (α) of .88. Item-scale correlations between items ranged from *r* = .51 to .76. Principal components analysis was performed and produced a single factor solution as expected (eigenvalue = 4.479, 56% of the variance explained). Factor loadings ranged from .60 to .84 among the 8 items.

**Table 1:** eHEALS scale reliability and factor analysis

**Item**	**FactorLoading**	**Mean Item-Total Correlation**
Q1: I know how to find helpful health resources on the Internet	.77	.68
Q2: I know how to use the Internet to answer my health questions	.79	.70
Q3: I know what health resources are available on the Internet	.77	.68
Q4: I know where to find helpful health resources on the Internet	.84	.76
Q5: I know how to use the health information I find on the Internet to help me	.81	.73
Q6: I have the skills I need to evaluate the health resources I find on the Internet	.72	.63
Q7: I can tell high quality from low quality health resources on the Internet	.65	.55
Q8: I feel confident in using information from the Internet to make health decisions	.60	.51
**Variance accounted for = 56%**		
Coefficient alpha = .88		

### Test-Retest Reliability

eHealth literacy scale scores were calculated and test-retest reliability was assessed by Pearson product moment correlation between scores at each interval (time 1 to time 4) using a standard regression model (SPSS REGRESSION) and using the intra-class correlation coefficient (SPSS RELIABILITY ICC MODEL (MIXED)). eHealth literacy scale scores were modestly correlated between administrations of the eHEALS, ranging from *r* = .49 to .68 (Table 2) . The intra-class correlation between the different scores was .49, suggesting that the eHEALS had modest stability over time.

**Table 2:** eHEALS test-retest reliability correlations

	eHealth Literacy Score Time 1	eHealth Literacy Score Time 2	eHealth Literacy Score Time 3	eHealth Literacy Score Time 4
eHealth Literacy Score Time 1	-			
eHealth Literacy Score Time 2	.68	-		
eHealth Literacy Score Time 3	.46	.49	-	
eHealth Literacy Score Time 4	.40	.40	.52	-

### Relationship Between eHEALS and Other Measured Variables

Baseline levels of eHealth literacy were higher among males (*t*_726_ = 2.236, *P* = .026); however, no statistically significant differences were detected in scores at post-intervention and 3 and 6 month follow-up administrations of the eHEALS. Age did not predict eHealth literacy scores at any time point. No significant relationship was found between eHealth literacy and use of information technology overall or with respect to any individual forms of technology surveyed (WWW, TV, instant messaging, email, pager, or mobile phone) (*P* = .05). eHealth literacy levels were also not related to overall self-evaluations of health and were not a significant predictor of perceived health status over time in this sample.

## Discussion

The eHEALS has shown promise as a measure of the concept of eHealth literacy as defined as a set of skills required to effectively engage information technology for health. The eHEALS’ high levels of internal consistency and modest test-retest reliability suggests that it has utility in examining eHealth literacy over time to both assess natural histories and evaluate eHealth literacy intervention outcomes. While tools exist that enable consumers to critically evaluate eHealth resources [24], there remains a dearth of instruments that assess consumers’ skills at using eHealth in general. Indeed, relatively few validated measures exist for most of the key literacy conditions within the eHealth literacy model (eg, science literacy, information literacy). Thus, it is imperative that future studies examine the links between perceived skills, eHealth use, and health behavior and health outcomes. Of those literacy tools available, most require significant time resources to administer and analyze. The eHEALS was designed for simple, easy administration and thus can be used on its own or incorporated with other measures of health as part of a standard health assessment battery in primary care or to support health promotion planning.

eHealth literacy promotion takes place within a larger learning context, thus it makes sense to develop partnerships with other groups working within other literacy sectors in validating the eHEALS in relation to other measures of literacy, social functioning, health, and well-being. Two examples of such multi-sectoral partnerships include the National Literacy and Health Program sponsored by the Canadian Public Health Association [25] and the Learners Advisory Network of the Movement for Canadian Literacy [26], which brings literacy groups together to address systemic literacy issues. Such partnerships illuminate the shared challenges in creating capacity for research, development, and policy advocacy around health and literacy issues.

### Limitations and Opportunities for Further Research

Conducting this study as part of a larger trial did pose problems for test-retest reliability; therefore, these results should be interpreted with caution. The lower than expected test-retest correlations between administrations of the eHEALS is attributed to a rise in eHealth literacy scores from baseline to post-intervention follow-ups, attributed to the smoking prevention intervention used in collecting the data [20]. Although unanticipated, one potential explanation for this increase is that the control intervention was designed based on the principles advocated by the eHealth literacy intervention itself (eg, user-friendly and audience-specific language, easy to read and navigate), which could have influenced participants’ eHealth literacy scores. This may explain the relatively modest correlations (.68 to .70) compared with what was expected.

Additional studies are required to longitudinally examine the eHEALS in study conditions that are not susceptible to influence of the characteristics of a specific intervention. Testing the eHEALS with a population that has high rates of information technology presents a limitation; however, it also provides an opportunity to understand the robustness of the measure within a specific population. Further research needs to consider the eHEALS’ application to other populations as well as groups with highly variable levels of technology familiarity.

The eHEALS measures consumers’ *perceived* skills and comfort with eHealth, not the skills directly. The eHealth literacy model includes six types of literacy, and thus each skill would require independent measurement, such as rigorous usability tests of standard computer equipment for computer literacy and reading aloud text passages to assess basic prose literacy. For health practitioners and consumers alike, such detailed assessment would be problematic in practice; however, it is worthwhile considering ways to conduct such measures in the future.

### Conclusions

The need for skills in seeking, appraising, and applying lessons learned through use of eHealth resources is common across ages, genders, and cultural groups, and thus the potential applicability of the eHEALS as a standard assessment tool for gauging eHealth literacy in health care is high. Assessing consumers’ comfort in using eHealth allows for the identification of skill gaps and can better assist those with low comfort levels in taking advantage of the potential benefits that eHealth can afford. Doing so may foster development of tools that can meet these needs and aid in creating appropriate strategies for bridging the digital divide in consumer health care quality. Only by increasing the understanding of the disparities between available eHealth tools and consumers’ abilities to use them can the necessary steps towards eliminating them be taken.

## Multimedia Appendix 1

eHealth Literacy Scale

## Abbreviations

eHEALS: eHealth Literacy Scale
